# Complementary Medicine Health Literacy among a Population of Older Australians Living in Retirement Villages: A Mixed Methods Study

**DOI:** 10.1155/2016/5672050

**Published:** 2016-06-26

**Authors:** Caroline A. Smith, Esther Chang, Suzanne Brownhill, Kylie Barr

**Affiliations:** ^1^National Institute of Complementary Medicine, Western Sydney University, Locked Bag 1797, Penrith, NSW 2751, Australia; ^2^School of Nursing and Midwifery, Western Sydney University, Locked Bag 1797, Penrith, NSW 2751, Australia

## Abstract

*Background*. Older Australians are consumers of high levels of complementary medicines. The aim of this study was to examine health literacy in a population of older Australians related to their use of complementary medicine.* Methods*. A two-phase sequential mixed method design incorporating quantitative and qualitative methods was used in this study. The first phase consisted of a cross-sectional survey using a validated health literacy questionnaire and follow-up interviews with 11 residents of retirement villages. Interviews explored low scoring domains on the health literacy questionnaire.* Results*. Health literacy competencies scored higher for the domains of* having sufficient information to manage their health; felt understood and supported by health care providers; actively managed their health; and having social support for health*. Three health literacy domains scored low including* appraisal of health information; ability to find good information;* and* navigating the health care system.* The findings suggest that participants had different experiences navigating the health care system to access information and services relating to complementary medicines. Two themes of “trust” and “try and see” provide insight into how this group of older Australians appraised health information in relation to complementary medicines.* Conclusions*. With a focus on self-care there is a need for improved health literacy skills.

## 1. Introduction

To be health literate implies having a range of skills and knowledge about health and health care, including the ability to find, understand, interpret, and communicate health information, seek appropriate care, and make critical health decisions [1]. Studies suggest that individuals with low levels of health literacy have less knowledge about their health condition, the treatments available, and the skills needed to negotiate the health care system [2]. Systematic reviews of relevant literature conclude that low levels of health literacy are associated with poorer treatment outcomes including poor compliance with medication, increased admissions to emergency departments, lower ability to interpret labels and health messages, reduced health status, and increased mortality among the elderly [3]. Health literacy has global significance across populations. Many international health and social policies highlight the importance of health literacy of individuals with the management of their health to achieve optimal, safe, and effective treatment outcomes [4, 5].

There has been significant discussion in the literature about what constitutes health literacy and how to measure it. The health literacy Ophelia Project undertaken at Deakin University, Australia, identified nine concepts of health literacy [6]. These include having sufficient information to manage health; social support for health; appraisal of health information; ability to engage with health care providers; navigating the health care system; ability to find good health information; and understanding health information well enough to know what to do with it. To date this model has not been examined in a complementary medicine or therapy (CM) context with any population group.

More than three million Australians (14% of the population) are aged over 65 years [7] and take an active role in their health care and personal responsibility for maintaining their health. There is growing evidence that this demographic is high users of CMs. Fifty-eight percent of those aged over 65 years have used one of 17 common CM modalities in the previous 12 months, and 65% of these had visited a CM provider [8]. The Australian Longitudinal Study of Aging, an ongoing prospective study of the older population, demonstrated the prevalence of CM utilisation to have increased over time ranging from 17% in 2000-01 to 35% in the 2003-04 period [9]. Data from the longitudinal survey indicate that celery, garlic, and ginkgo biloba are the most common herbal medicines used, and cod liver oil is the most popular nutritional supplement [9]. CM is generally used to treat a wide range of chronic health complaints that become increasingly common with age [10], particularly musculoskeletal conditions and pain [11] and anxiety or depression [12]. Research clearly highlights that older Australians experience significant benefits to their health and well-being from CM and that it is highly valued. Complementary health care may offer a way for older people to cope with their ill health or to engage in maintaining their health.

However, use of CM without adequate supervision by a qualified health practitioner can be of concern among an older population, due to a higher prevalence of polypharmacy arising from the treatment of complex chronic health conditions [13]. The elderly may also be more susceptible to medication sensitivity due to less optimal organ function associated with aging [14–16]. Together these concerns may increase the risk of potential CM-drug interactions. In the Australian community the prevalence of serious adverse reactions to CM is relatively low compared to pharmaceutical medications [13]; however mild reactions are more common. A retrospective review of previously collected health data analysed the outcomes of the 15% of individuals who reported using CM. 5.8% were identified as having a significant risk of an adverse reaction [17]. These risks were linked to garlic, ginkgo, and combinations with drugs affecting blood coagulation, such as aspirin, representing 95% of the list of significant interactions. Other higher risk CM product and drug combinations identified as potentially dangerous included garlic or ginkgo with warfarin, the combination of ginseng and warfarin, and the combination of St. John's wort with digoxin.

This risk of interactions is complicated by low disclosure of CM use between consumers and their health care providers. A study by Braun et al. identified many people who experience a suspected adverse reaction to CM do not inform their health care provider and choose to self-manage their reaction [18]. A review of studies highlights the rate of nondisclosure among those using CM to their health care providers can be as high as 60–70% [19]. The reasons for this nondisclosure are varied and can include the individual forgetting to mention CM use, disclosure not being seen as relevant, the doctor not asking about CM use, and the doctor not respecting the value of CM. Disclosure and communication about CM are essential for achieving optimal treatment outcomes. These findings suggest that health literacy in an elderly population of CM users may be poor. The aim of this study was to examine health literacy in a population of older Australians currently using CM.

## 2. Methods

A two-phase sequential research design incorporating both the quantitative and qualitative (mixed) methods was used in this study. Qualitative research provides new knowledge (inductive) about the health literacy scores generated from quantitative methods (deductive). Both methods increase our understanding of the experiences and behaviour of older Australians related to their health literacy and use of CM (the phenomenon under study) and inform and give meaning to the low scoring constructs of the Health Literacy Questionnaire (HLQ). The first phase, a cross-sectional survey, used the validated HLQ, and the second phase involved the follow-up interviews with study participants selected from the survey. Ethics approval was obtained from Western Sydney University Human Research Ethics Committee (H10520); participant informed consent was obtained. The study was implemented between April 2014 and February 2015.

### 2.1. Participants

Men and women aged 65 years and over were recruited from retirement villages and health care facilities in the Greater Western Sydney Region and the Southern Highlands of New South Wales, Australia. In Australia a retirement village is community comprising housing for people aged over 55 years, residents live independently, and many villages may offer some health care services, leisure facilities, and social clubs. The village provides smaller, manageable housing, supportive of the changing needs of older people. There are more than 1,800 retirement villages in Australia. Approximately one-third are located in New South Wales, accommodating over 100,000 residents. Jones Lang LaSalle estimated that in 2008-2009 about 5% of the Australian population over the age of 65 were living in retirement villages [20]. The entry age of residents is between 71 and 83 years, and many are attracted to the lifestyle benefit offered by retirement villages. This setting is different to a residential aged care facility which can provide seniors with residential living environment where nursing support is provided for their day-to-day lives. This can range from a little assistance through to full palliative care.

The selection criteria for the study included participants who were current users of CM, defined as use in the previous 12 months and an ability to read and write English. In the absence of a sampling frame (identifying CM users) we mapped out retirement villages and aged care facilities with independent living facilities and wrote to the managers of 23 sites. We received responses from 16 sites agreeing to participate in and promote the study. At each site the study was promoted using posters, flyers, and newsletters inviting village residents to attend a presentation on the study by members of study team.

### 2.2. Phase  1: Cross-Sectional Survey

The survey utilised the self-administered HLQ. The validated HLQ [6] consists of nine scales and 55 items. The scales cover both intrinsic and extrinsic dimensions of health literacy. Some scales reflect the individual's capability to (a) understand, engage with, and use health information and health services and (b) reflect the capability of an organisation to provide services that enable a person to understand, engage with, and use health information. The nine scales include feeling understood and supported by health care providers; having sufficient information to manage an individual's health; an ability to actively manage an individual's health; social support for health; appraisal of health information; ability to actively engage with health care providers; navigating the health care system; ability to find good health information; and understanding health information well enough to know what to do. One scale, “ability to find good information,” consists of five items:

1. Find information about health problems.
2. Find health information from several different places.
3. Get information about health so you are up to date with the best information.
4. Get health information in words you understand.
5. Get health information by yourself.

A high score on this scale describes a person as an information explorer and someone who actively uses a diverse range of sources to find information and is up to date. A low score on this scale describes people who cannot access health information when required and is dependent on others to offer information. Each scale includes 4 to 5 items, with participant's indicating their response along a Likert Scale with response options ranging in “1 = very difficult, 2 = difficult, 3 = easy, and 4 very easy” or along a five-point scale ranging from “strongly agree to strongly disagree.” The HLQ has strong psychometric properties being grounded in the individual's lived experience and is validity driven [6]. Reliability testing was examined using Raykovs procedures rather than Cronbach's alpha where >0.80 was sought. This was achieved for eight of the nine scales; the lowest reliability estimates were achieved for the domain appraisal of information (0.77).

Participants were asked to respond to the HLQ recalling their experience and use of CM, for example, consultation or discussion of CM with a health professional or CM health professional and prescribing and dispensing of a CM product or delivery of a treatment, to their interaction with a pharmacy assistant and the purchase of an over-the-counter CM product. In addition, data on CM use and demographic information were collected including age, gender, current living arrangement, and education status. Our definition of CM included those treatments described as self-care or care delivered by CM practitioners.

### 2.3. Analysis

Statistical analysis was carried out using SPSS software (the IBM Statistical Package for the Social Sciences 19, Chicago, IL). Descriptive statistics, using frequencies and percentages, were calculated for demographic and categorical data to describe the population. Health literacy scales were calculated using a scoring algorithm for the HLQ version 1 (dated 2012). The algorithm produces unweighted scale scores for the nine scales of the HLQ, with the final score for each subscale, an average score across all items forming the scale. For missing values this program uses an algorithm to impute missing values. For scales with 4 to 5 items two missing values can be imputed. For scales with 6 items 3 missing values can be imputed, and if more responses among the scale items were missing, scale score was not computed. Scores were summarised with reporting of mean and standard deviation.

### 2.4. Phase  2: In-Depth Interviews with Participants Selected from the Sampling Frame of the Survey

The purpose of the interviews was to explore CM health literacy constructs from the HLQ that scored low, appraisal of health information, ability to find good information, and understanding of health information sufficiently to know what to do. The sampling frame for the qualitative phase of the study was formed by study participants who consented to participate in the interview. A sample of participants was sought through theoretical and purposive sampling to ensure a socially diverse sample of participants across demographic variables such as age, gender, education, and low scoring scales of the HLQ.

A telephone or face-to-face in-depth interview was conducted with the participants, and both modes of interview were digitally recorded. Information was presented to participants about the survey and the follow-up interviews in the participant information sheet. The majority of interviews [8] were conducted face-to-face in the participants' home, and for some interviewees they may have had their spouse or carer in the same room. Three interviews were conducted over the telephone. No preexisting relationship existed between the participant and interviewer. This relationship was established when contact was first made when arranging the interviews. The research assistant who undertook the interviews (KB) had two-year research experience working in the CM field and was undertaking research training as part of an honours undergraduate degree. The first three interviews were checked for accuracy and consistency, and minor adjustments were made to the interview guide and used for the remainder of the interviews.

Participants were not made aware of the specific questions to be explored in the interview other than some question responses to the questionnaire which would be explored in greater detail. Each interview took approximately one hour and was undertaken by the research assistant (KB). No repeat interviews were conducted, and when no new data were obtained that contributed to emerging themes recruitment of additional participants ceased. Interview transcripts were not returned to participants for comment. The interviews were transcribed verbatim. Following repeated readings, data were coded with reference to the three low scoring scales of health literacy, for example, appraisal of health information, ability to find good information, and understanding of health information sufficiently to know what to do. Further coding of data occurred along with identification of linkages and relationships between themes, using thematic analysis [21].

## 3. Results

A total of 82 questionnaires were completed by participants residing in 16 retirement villages in South West Sydney and the Southern Highlands of New South Wales. Ten questionnaires were subsequently removed from the database due to non-CM use by study participants. The 72 remaining study participants were mostly women, aged in their late 1970s, who were living alone, were born in Australia or New Zealand, completed high school, and had health private insurance (Table 1). Participants were living with complex health conditions, arthritis, and back pain being the most common conditions, and 25% lived with a current heart condition. The precise numbers of participants meeting our study criteria at each village were unknown. We are unable to report a response rate. This could not be determined due to the multifunction of the living environments included in some retirement village settings, which were also combined with residential aged care. However, the demographic characteristics of the 10 study participants who were non-CM users indicate similar age, gender, country of birth, English spoken at home, and arthritis and health complaint characteristics to CM users.

Participants were asked about their current CM use. Seventy-two currently used CM, and our data indicates participants used more than one CM method in the last 12 months (61, 84.7%) (Table 2). The most commonly used CMs were biologically based, for example, vitamins and minerals. Eleven participants reported taking a combined multivitamin supplement alone, whilst others reported taking a combination of single vitamins or a combination of a single vitamin in addition to a combined multivitamin. Vitamin D alone was the most commonly used single-use vitamin (33, 45%), followed by vitamin C (15, 20.8%). Mineral supplementation was commonly reported, for example, calcium (23%) and magnesium (10, 13.8%). Biological products and supplements were also used including glucosamine 17% and fish oils used by a third of participants. Use of herbal medicines was reported by 25%. Body based practices were used less frequently including massage (17, 23.6%), chiropractic and osteopathic techniques (10, 13.8%), relaxation (8, 11.1%), meditation (12, 16.7%), tai chi (10, 13%), and yoga (4, 4.2%).

### 3.1. Health Literacy

Six scales of the HLQ scored greater than 3.0 (Table 3). Participant's responses indicated agreement with scale items:* having sufficient information to manage my health; feeling understood and supported by health care providers; actively managing my health; *and* having social support for health*. The following tasks were rated as “easy to do”:* ability to actively engage with health care providers* and* understand health information well enough to know what to do*. Three HLQ scales were identified as having less competencies or identified as “difficult to do”: appraisal of health information (2.9 out of possible score of 4), ability to find good information (3.8 out of score of 5), and navigating the health care system (3.8 out of 5). The relationship between the health literacy domains and demographic data was examined; however no associations were found (data not shown).

### 3.2. Qualitative Results

Eleven participants were interviewed including nine women aged between 65 and 83 years and two men aged 80 and 92 years. Women were users of higher levels of CM compared with men. In particular, three women in the group used multiple types of biological products and practitioner services for various health conditions (e.g., multivitamins, chiropractic, massage, minerals, tai chi, osteopathy, homeopathy, and relaxation). Men reported accessing and using minerals and biological products (i.e., calcium, vitamins, and fish oil). The gender, age, country of birth, language spoken at home, and education were similar to those participating in the survey (Table 1), although those interviewed reported less chronic disease.

#### 3.2.1. Navigating the Health Care System

Participants had different experiences navigating the health care system with responses focusing on finding the right support in relation to CM, sourcing the best treatment, and getting to see the health provider they needed:

*it wasn't difficult…I had been to the doctor and I said to him I am in pain and he said well you have got inflammation but he didn't give me anything…I went to work… and ….James said to me why don't you try an acupuncturist and that's how easy it was so I did. (female, 07) *

A participant reported that she was challenged by a doctor about going to see the chiropractor. She decided not to inform him about her visits, “*I just thought what you don't know won't hurt you. I'll keep going because I find benefit from it. If I hadn't been getting any benefit from it, I would have stopped” (female, 08)*.

A trusted source was identified as a means to access CM. Participants also differentiated between the perceived role of retail outlets:

*I do think people take a lot of risks buying over the counter, and they need to get some sort of referral from a person they trust. It maybe their doctor or a complementary person but you don't just go in and buy stuff haphazardly. You can do yourself a lot of harm because some things don't agree with other things. (female, 09) *

*I have tried not going to the pharmacies but to the chemist warehouses and things like that but I mean really all they want to do is sell as much as they can, so I leave those alone as a rule. (female, 06)*

#### 3.2.2. Ability to Find Good Information

Participants said that it was important to find good information about CM. Participants appear aware of the risk of side effects and highlighted this topic as a key area for sourcing good information. Participants reported using books, talking to health professionals, and searching for information on the internet:

*I think people need to be careful what they take and who recommends them to take whatever because so much stuff is advertised and they say its good for this and wonderful for that…. (female, 09)*

* I want to know the side effects (participant 06)… side effects if any, what they do and don't interact with. (female, 08)*

*I just wish there was more leaflets or something given out, I mean there's all these vitamins and minerals advertised and half, you know, lots of us really don't know which one is which. (female, 05) *

#### 3.2.3. Appraisal of Health Information

Two themes captured how people appraised health information in relation to CM, “trust” and “try and see.” 

*Trust*. Participants described how trust influenced their decision-making. Trusting information was influenced by reading advertising material and seeking information about detrimental side effects reported from the products or questioning their doctor to receive more objective information:

*I could say that I'm really impressed with the current advertising procedures because they present glowing reports of such and such a treatment or whatever, but I suppose I'm a bit suspicious by nature. …Not to say that they're not true for some people, or the results aren't true for some people and so on, but the glowing reports may not tell you all the pros and cons of the results. (male, 03)*

*Well basically if I've got any queries about a particular thing I think, how do I trust this? Usually the manufacturer of the particular item will have a website, so I will go that website and then I look up the information on that. And you'll get people's reactions to it you know, and they'll say I've tried this product, it worked excellent for me, tried this product, didn't work for me. So I weigh out the pros and cons from that. (female, 08)*

*Try and See*. Other participants were less analytical in their approach to evaluating information to guide their decision to use CM. Some participants used information from one source and did not compare information from several sources. Their use of information suggests that they relied on word of mouth from others who had used a product, and where it has worked one study participant decided that was good enough for her to consider use of CM:

*Well, when you google it, you know, they come up with various things, you can look at them, and I have a look at maybe two. I don't go through the whole eight or nine pages of it. And I just pick one that I think, that sounds all right. (female, 06)*

*I think information from people who have used a product and found that it's worked for them, and then if you get a multitude of those people, you know, a reasonable sample, saying this is really good, I think it's certainly worth looking at. (female, 04)*

## 4. Discussion

Older Australians using CM living in retirement villages were making decisions regarding managing their own health. Our primary findings suggest that this population demonstrated competencies relating to health literacy; however, the variability around scores indicated that not all individuals have the same health literacy skills. We identified three scales that scored lower:* navigating the health care system; an ability to find good information; *and* appraisal of health information*. Interpretation of these scales suggests that for some participants no matter how hard they tried they could not understand most health information and were confused when there was conflicting information. They were also not able to access health information when required and were frequently dependent on others to offer information, unable to advocate on their own behalf, and unable to find someone who can help them use the health care systems to address their health needs. Findings from the interviews offered some explanations to why health literacy was lower and highlighted a lack of support and communication by some health professionals, a lack of information and concern relating to side effects and potential interactions impacting on the ability to make informed decisions, and the skills to appraise information resulting in a* try and see* approach.

Our data identified participants were challenged with finding good information and navigating the health system to access information and CM services. This observation is not new; findings from Salter et al. found that people expected to supplement their consultations with health professionals with their own searching of written information, media reports, and internet searches on CM [22]. Salter et al. also found that the vast amount of information available was not helpful or relevant and to make sense of this information help was sought from health professionals. Other studies indicate that communication barriers remain and can be impeded by doctors' dismissive attitudes, lack of interest, or time available to discuss CM [21, 23, 24]. Such attitudes remain unhelpful with addressing health literacy to facilitate informed decision-making regarding CM use.

Participants spoke about the barrier to obtaining good information and their attempts to appraise and make sense of information from multimedia sources including the radio, television, the internet, and books, as well as health professionals and family and friends. Our findings are similar to observations by Verhoef et al. who found that CM users based their considerations on information from “experientially based evidence, such as anecdotes, personal experience, and intuition,” rather than an external source of knowledge to inform their decisions regarding their choice of CM use [25]. The ability to find good information and appraise information became fraught with the large quantities of information available using the internet. An increasing number of adults older than 65 years are seeking information on the internet [26], with 38% of seniors using the internet; however the majority (75%) do not consistently check the source and date of information found. Responses from interviews also indicated that the information on the internet frequently influenced CM decision-making, and Shreffler-Grant et al. have identified that many of these sites make unsubstantiated claims to treat, prevent, diagnose, and/or cure specific diseases [27]. Low health literacy around these domains highlights the potential for older vulnerable people to be misinformed and to make inappropriate self-care decisions.

The use of CM ingestible products and supplements in our study confirms data and uses patterns from other studies [9, 10]. Older populations have been identified as of particular risk in relation to health literacy [18], and our findings have important implications for the safe and effective use of self-care management. Glucosamine, a nutritional supplement, was used by some study participants for osteoarthritis. This supplement has been well researched but the evidence for its effectiveness is mixed. A recent review and analysis of all the evidence show that, overall, there is little clinical benefit in terms of pain or changes in the joint [28]. Our health literacy findings could be considered conflicting when considering the evidence for glucosamine. This example demonstrates that health literacy is complex and relates not only to their health literacy abilities but also to their health care needs. Translation of research evidence is dependent on effective communication targeting consumers' needs and the many stakeholders providing health care. There is a need for balanced information communicated to the public of the risks and benefits of CM, but as our findings highlight there are gaps in CM health literacy that need to be addressed. One starting point could involve addressing the health literacy domains of navigating the health care system, an ability to find good information, and appraisal of health information.

A strength of our study includes the participants from urban and rural settings and from different culturally and linguistically diverse backgrounds. Use of mixed methods has been demonstrated to be useful in health care research [29], and our interview data provided a greater understanding and explanation of why health literacy scales such as navigating the health care system, the ability to find good information, and appraisal of health information constructs scored low.

The study does have some limitations. We were unable to report on the response rate to the survey and are uncertain of how generalisable the findings are to a wider older population of CM users. However, comparing the demographics of our study population with national census data of residents living in retirement villages demonstrates a similar age between these populations (mean 79.3; SD 4.3) [30] and our findings may be applicable to other residents in retirement villages. We approached organisations initially by email or letter and this may have resulted in a potential sample bias arising from an initial screen from the organisation, leading to participation by organisations specifically interested in CM. We used announcements in newsletters and mailings via the host organisation; this may have led to an overrepresentation of older Australians who have higher health literacy and more experience and confidence in using CM. Interviews with participants focused on gaining more in-depth understanding of health literacy relating to specific demographics and current CM users; these may not reflect the broader population of older Australians using CM. It is also possible that during the interviews participants may have been reluctant to be too critical. Further research with larger populations is needed.

## 5. Conclusion

The study population demonstrated competencies in some health literacy domains. However health literacy scales including navigating the health care system; an ability to find good information; and appraisal of health information highlight a need to develop health literacy skills among older Australians who were using CM. Our findings have the potential to inform an educational intervention to address these gaps including developing skills to identify good and reliable sources of information and to resolve conflicting information and the ability to access and use a diverse range of information sources to find information that is up to date that can be used to guide their decision with their health care providers. Improved health literacy will enhance appropriate use of health services and ultimately enable older Australians to engage in taking an active role in their health and reducing the potential for adverse health outcomes.

## Figures and Tables

**Table 1:** Sociodemographics of responders.

Demographics *n* = 72	*n*	%	Interviewed participants		Residents ineligible	
Sex						
Male	20	27.8	2	18.2	3	30.0
Female	51	71.8	9	81.8	7	70.0
Missing	1	1.4				
Age^*∗*^	78.9	6.55	78.2	6.9	79.5	8.6
Do you live alone						
Yes	36	50.0	2	18.1	4	40.0
No	29	40.3	5	45.4	5	50.0
Missing	7	9.7	4	36.3	1	10.0
Country of birth						
Australia	48	66.0	8	72.7	7	70.0
Europe (UK 14, Estonia 3, Holland 2, Ireland 1)	20	27.7	3	27.2	3	30.0
Others	4	5.5				
Speak English at home						
Yes	66	91.7	11	100.0	10	100
No	3	4.2				
Missing	3	4.2				
Highest level of education						
Primary school or less	3	4.2	0	0.0		
High school (not completed)	13	18.1	1	9.0	3	30.0
High school (completed)	25	34.7	7	63.6	3	30.0
TAFE/trade	13	18.1	2	18.1		
University	14	19.4	0	0.0	4	40.0
Missing	4	5.6	1	9.0		
Private health insurance	43	59.7	7	64.0	8	80.0
Have a health care card	52	72.2	10	90.9	9	90.0
Current health status						
Arthritis	41	56.9	5	45.5	6	60.0
Back pain	35	48.6	6	54.5	3	30.0
Heart problems	21	29.2	4	36.3	1	10.0
Cancer	11	15.3	0	0.0	0	0
Depression/anxiety	11	15.3	1	9.0	1	10.0
Diabetes	10	13.9	1	9.0	0	0.0
Asthma	8	11.1	0	0.0	2	20.0
Stroke	6	8.3	1	9.0	1	10.0

^*∗*^M: mean; SD: standard deviation.

**Table 2:** Current complementary medicine and therapy use.

CM modality *n* = 72	*n*	%
Mineral supplements		
Calcium	17	23.6
Magnesium	10	13.8
Biological products/supplement (defined as substances found in nature, such as herbs, foods, and vitamins)		
Multivitamin	11	15.3
Individual vitamins		
A	4	5.6
B	6	8.3
C	15	20.8
D	33	45.8
E	4	5.6
K	1	1.4
Fish oils	24	33.3
Glucosamine	12	16.6
Specific vitamin (C, E) and mineral supplement (copper and zinc)	7	9.7
Herbal medicines	18	25.0
For example, slippery elm, cranberry, saw palmetto		
Massage	17	23.6
Acupuncture/acupressure	12	16.7
Tai chi/Qigong	10	13.8
Meditation	12	16.7
Chiropractic/osteopathy	10	13.8
Relaxation	8	11.1
Yoga	4	4.2
Others (aromatherapy, Bach flower remedies, magnets, homeopathy)	17	23.6

**Table 3:** Participant response to the health literacy scales of the HLQ.

Health literacy scale	*n*	M	SD
How strongly do you disagree or agree (1–4 point scale)			
Having sufficient information to manage my health	69	3.20	0.54
Feeling understood and supported by health care providers	71	3.19	0.52
Actively managing my health	71	3.14	0.44
Social support for health	71	3.00	0.54
Appraisal of health information	69	2.90	0.49
How difficult or easy the following tasks are for you now (5 point scale cannot do to always easy)			
Ability to actively engage with health care providers	69	4.03	0.62
Understand health information well enough to know what to do	70	4.00	0.58
Ability to find good information	70	3.84	0.65
Navigating the health care system	71	3.88	0.61

M: mean; SD: standard deviation.

## Abbreviations

CM: Complementary medicine or therapy
HLQ: Health literacy questionnaire.
